# Zn-Al Ferrite/Polypyrrole Nanocomposites: Structure and Dielectric and Magnetic Properties for Microwave Applications

**DOI:** 10.3390/polym16172432

**Published:** 2024-08-28

**Authors:** Huda F. Khalil, Sherif G. Elsharkawy, Nouf F. AL-Harby, Mervette El-Batouti

**Affiliations:** 1 Electronic Materials Department, Advanced Technology and New Material Institute (ATNMI), City of Scientific Research and Technological Applications (SRTA-City), Alexandria 21934, Egypt; 2 Basic and Applied Sciences, College of Engineering and Technology, AASTMT, Alexandria 21934, Egypt; ssharkawy@aast.edu; 3 Department of Chemistry, College of Science, Qassim University, Buraydah 51452, Saudi Arabia; 4 Chemistry Department, Faculty of Science, Alexandria University, Alexandria 21934, Egypt; mervette_b@yahoo.com

**Keywords:** Zn-Al ferrite, polypyrrole, nanocomposites, magnetic properties, dielectric properties, microwave absorption, impedance spectroscopy, core–shell structure

## Abstract

In this study, Zn-Al ferrite/polypyrrole (PPy) nanocomposites were synthesized and thoroughly characterized to explore their potential for microwave applications. X-ray diffraction analysis confirmed the presence of ZnO, AlFeO_3_, and Fe_2_O_3_ phases, with the crystal size decreasing from 31 nm to 19.6 nm as aluminum content increased. High-resolution transmission electron microscopy (HR-TEM) revealed a distinctive core–shell morphology, where the polypyrrole encapsulates the ZnAl*_x_*Fe_2−*x*_O_4_ particles. Magnetic measurements showed that decreasing aluminum concentration led to a reduction in both saturation magnetization (Ms) from 75 emu/g to 36 emu/g and remanent magnetization (Mr) from 2.26 emu/g to 2.00 emu/g. Dielectric analysis indicated that both the real (ε′) and imaginary (ε″) components of dielectric permittivity decreased with increasing frequency, particularly between 10 and 14 GHz. Furthermore, electrical modulus analysis highlighted the significant impact of aluminum doping on relaxation time (τIP), indicating the presence of interface polarization. Impedance spectroscopy results underscored the dominance of interface polarization at lower frequencies and the presence of strong conduction paths at higher frequencies. These combined magnetic and dielectric loss mechanisms suggest that the Zn-Al ferrite/polypyrrole nanocomposite is a promising candidate for advanced microwave absorption applications.

## 1. Introduction

Microwave technology has become increasingly important in various applications, including telecommunications, radar systems, and energy transmission. However, the efficiency of microwave energy utilization remains a critical challenge. Many researchers have made efforts to develop advanced microwave-absorbing materials that can be used in the field of microwave applications [1]. Nanocomposites have been raised among these materials, combining different elements for more refined and improved performance. Then, further investigations have been made to include polymers in addition to these nanocomposites, for example, Mn-Zn ferrites [2], Cu-Zn ferrites [3], ZnFe_2_O_4_ [4], and Zn_0.5_Ni_0.4_Cr_0.1_Fe_2_O_4_ [5], all these nanocomposites are fabricated by combining the ferrite with conductive polymers like polyaniline (PANI) and polypyrrole (PPy). These nanocomposites have shown excellent microwave absorption performance, which is attributed to the effects of magnetic and dielectric losses.

Throughout this work, spinel ferrites, such as zinc–aluminum (Zn-Al) ferrite, have been given significant attention due to their excellent dielectric and magnetic properties, making them suitable for microwave absorption applications [6,7]. Polypyrrole, PPy [8], has been widely used with various ferrites to increase the magnetization saturation (Ms), and their ability to absorb electromagnetic waves makes them suitable for shielding electronic devices from unwanted interference. Accurately mixed ferrite powder with PPy produces nanocomposites characterized by highly dielectric and magnetic properties. Understanding the interplay between the spinel ferrite and conductive polymer components will contribute to the development of advanced microwave-absorbing materials, ultimately leading to improved efficiency and performance in various microwave-based technologies. The improved structure of the Zn-Al ferrite/PPy nanocomposite leads to better electromagnetic interference (EMI) shielding performance [9]. Moreover, this structure constitutes various applications, including radar stealth technology, wireless communication systems, and electromagnetic compatibility. In this work, the microwave absorption properties of the Zn-Al ferrite/PPy nanocomposite will be explored, and the potential for effective microwave absorption and shielding in diverse microwave-based applications will be discussed. This will be achieved by investigating the structural, dielectric, and magnetic properties of Zn-Al ferrite/PPy nanocomposites, with the goal of advancing the efficiency of microwave applications. The research will focus on the synthesis, characterization, and evaluation of the nanocomposites’ performance, providing insights into the underlying mechanisms responsible for their enhanced microwave absorption capabilities.

## 2. Experimental Techniques and Procedures

ZnAl*_x_*Fe_2−*x*_O_4_/polypyrrole nanocomposite with concentrations of *x* (wt.%) = 0.0, 0.2, 0.4, 0.6, and 0.8 were synthesized using solid-state reaction technique [8]. The zinc oxide (ZnO), aluminum oxide (Al_2_O_3_), iron oxide (Fe_2_O_3_), and polypyrrole (PPy) powders were weighed according to the desired composition ratio (wt.%) for each sample. ZnO, Al_2_O_3_, and Fe_2_O_3_ powders were obtained from Sigma-Aldrich; also, PPy was synthesized using a chemical polymerization method. A calculated amount of PPy powder, approximately 30% of the total weight of the nanocomposite, was added to the weighed ZnO, Al_2_O_3_, and Fe_2_O_3_ powders. Then, the mixture was further ground to evenly distribute the PPy within the ZnAl*_x_*Fe_2−*x*_O_4_ matrix with ball milling. Finally, the mixture was calcinated at 850° for 7 h. After the calcination process, the resultant nanocomposite powder was removed from the furnace and allowed to cool down to room temperature. The synthesized ZnAl*_x_*Fe_2−*x*_O_4_/PPy nanocomposite was characterized using X-ray diffraction (XRD) using X’Pert Philips (Eindhoven, The Netherlands) powder diffractometer with CuKα radiation (λ = 1.54056 Å) in the range 10° ≤ 2*θ* ≤ 80°. Additionally, the morphology of the specimens was investigated using High-Resolution Transmission Electron Microscopy (TEM), JEOL-JEM-2100 (Tokyo, Japan) equipment was operated at 200 kV, and included a SAED (Selected Area Electron Diffraction) mode. The magnetic hysteresis loops were measured at room temperature using a vibrating sample magnetometer (VSM) operating system 7400 Series-Lake Shore Cryotronics (Westerville, OH, USA). The frequency-dependent electromagnetic properties and microwave shielding measurements were carried out on a vector network analyzer (model 8510–45 MHz to 26.56 GHz, USA) by waveguide transmission line technique. A vector network analyzer (VNA) for the microwave measurements was used. The measurements were carried out using the free space method, which is ideal for minimizing interference from external sources and ensuring accurate results. The configuration involved positioning the sample between two horn antennas in a free-space environment to measure the S-parameters (S11 and S21). The S-parameters were then used to extract the complex permittivity (ε) and permeability (μ) of the material. The extraction was performed using the Nicolson–Ross–Weir (NRW) method, a well-established technique for determining electromagnetic properties from S-parameter measurements. All instruments and methodologies used in this study were carefully selected to ensure high precision and reliability of the results.

## 3. Results and Discussion

### 3.1. XRD Analysis

**Figure 1:**
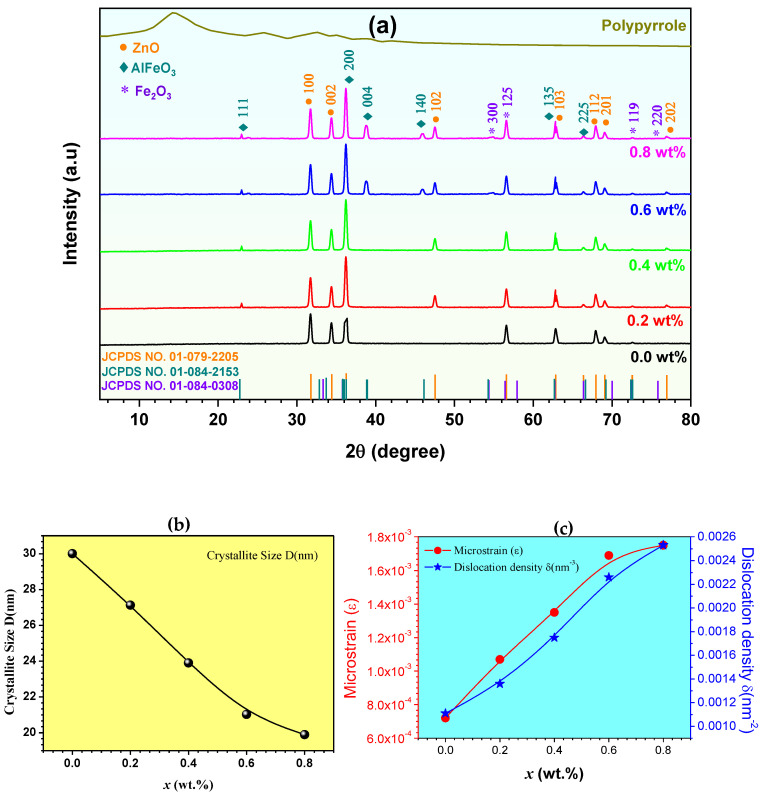
(**a**) XRD of ZnAl*_x_*Fe_2−*x*_O_4_/polypyrrole nanocomposite with concentrations of *x* (wt.%) = (0.0, 0.2, 0.4, 0.6, and 0.8). (**b**) crystallite size D (nm), (**c**) microstrain (ε), and dislocation density δ (nm^−3^) of the nanocomposite.

The XRD peaks of ZnAl*_x_*Fe_2−*x*_O_4_/polypyrrole nanocomposites with concentrations of *x* (wt.%) = 0.0, 0.2, 0.4, 0.6, and 0.8 are shown in Figure 1a. The XRD of the nanocomposite with *x* = 0.0 (wt.%) showed strong diffraction at 2*θ* = 31.82°, 34.21°, 36.33°, 47.49°, 56.52°, 62.90°, 67.95°, 69.01°, and 78.32°, corresponding to 100, 002, 200, 102, 125, 103, 112, 201, and 202 planes, respectively. The nanocomposite structure of Zn-Al ferrite/PPy contains three phases: the ZnO hexagonal phase (JCPDS No: 01-079-2205), AlFeO_3_ orthorhombic phase (JCPDS No: 01-084-2153), and Fe_2_O_3_ rhombohedral phase (JCPDS No. 01-084-0308). Figure 1b illustrates the crystallite size (D) nm, and Figure 1c shows the lattice microstrain (ε) and dislocation density (δ)nm^−3^ for ZnAl*_x_*Fe_2−*x*_O_4_/polypyrrole nanocomposites with concentrations of *x* (wt.%) = 0.0, 0.2, 0.4, 0.6, and 0.8. The corresponding results are tabulated below in Table 1 and were obtained using the following formulae [10,11]:

(1) $$D=\frac{k\lambda }{\beta cos\theta }$$

(2) $$\varepsilon =\frac{\beta \cos\theta }{4}$$

(3) $$\delta =\frac{1}{D^{2}}$$

where (*k*) is the Scherer’s constant, typically taken as 0.89; (*λ*) is the wavelength of the X-rays; (*β*) is the full width at half-maximum (FWHM) of the diffraction peak; and *θ* is the Bragg’s angle. From Figure 1b, the crystallite size, *D*, was observed to decrease from 30.01 to 19.89 nm as the Al^3+^ substitution ratios *x* (wt.%) were increased from 0.0 to 0.8, showing a 66.27% loss in the total crystallite size. This reduction in size could be referenced to the disruption of the crystal growth process due to the differing atomic size, bonding, and diffusion characteristics of the substituted Al^3+^ ions compared to the original Fe^3+^ ions. The inhibition of the diffusion of atoms necessary for continued crystal growth is caused by the altered atomic interactions and mobility introduced by the Al^3+^ substitution. Moreover, the crystal structure destabilizes and hinders long-range crystal order due to the incorporation of smaller Al^3+^ ions. The increased lattice strain and defects introduced by the size mismatch between the substituted Al^3+^ and original Fe^3+^ ions interrupts the normal crystal nucleation and growth mechanisms. The presence of the Al^3+^ ions disrupts the regular crystal formation processes [12,13]. Doping can alter the nucleation and growth kinetics of crystallites, leading to variations in size. Conversely, the lattice microstrain and dislocation density exhibited an opposing trend, as illustrated in Figure 1c. Certainly, the opposing trend observed suggests an intriguing aspect of the material’s behavior. This can be attributed to the introduction of Al^3+^ as a dopant; the incorporation of foreign atoms into the crystal lattice may cause lattice distortion or mismatches, contributing to an increase in lattice strain [14,15].

### 3.2. Morphology

**Figure 2:**
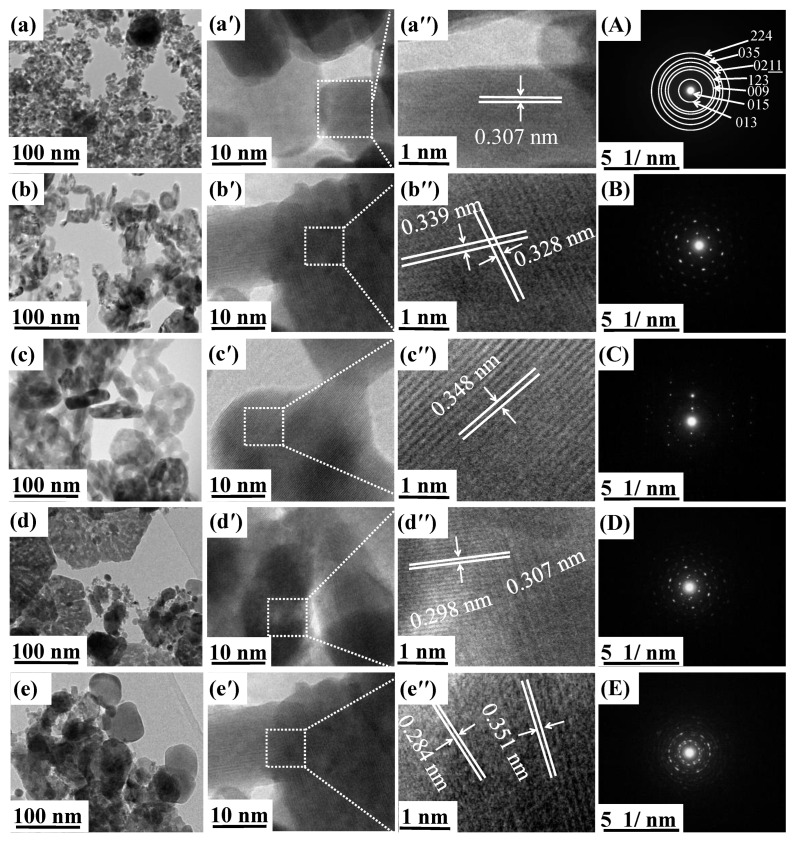
HR-TEM images of ZnAl*_x_*Fe_2−*x*_O_4_/polypyrrole nanocomposite with concentrations of *x* (wt.%) = 0.0, 0.2, 0.4, 0.6, and 0.8, (**a**–**e**) alignment 100 nm, (**a′**–**e′**) alignment 10 nm, (**a″**–**e″**) alignment 1 nm, and (**A**–**E**) SEAD.

The morphology of the ZnAl*_x_*Fe_2−*x*_O_4_/polypyrrole composite powders was characterized using transmission electron microscopy (TEM), as shown in Figure 2a–e. For each compound, a TEM image (100 nm scale), an HR-TEM image (10 nm scale) (see Figure 2a′–e′), another HR-TEM image (1 nm scale) (see Figure 2a″–e″), and a SAED diagram (see Figure 2A–E) are presented. TEM patterns show a significant difference in particle shape upon the substitution of Zn for Al in the spinel framework. Figure 2a,d show that most of the ZnAl*_x_*Fe_2−*x*_O_4_ particles at concentrations of *x* = 0.0 and 0.6 are faceted with a hexagonal shape [16]. These nanoparticles adopted a well-defined polyhedral morphology with an alignment of 100 nm. This implies that the high calcination temperature (850 °C) not only affects the shape but also the size of the particles. However, in Figure 2e, primary particles have irregular shapes and are agglomerated, a morphology inside the composite. This indicates that the coexistence of the ZnAl*_x_*Fe_2−*x*_O_4_ spinel and the secondary Fe_2_O_3_ phase results in a polycrystalline sample with a large proportion of irregularly shaped particles, where HR-TEM Figure 2b″,d″ enlarged from the selected area show lattice fringes with interplanar distances of 0.307, 0.339, and 0.348 nm for ZnAl_0.2_Fe_1.8_O_4_, ZnAl_0.4_Fe_1.6_O_4_, and ZnAl_0.6_Fe_1.4_O_4_ samples, respectively. These match well with the (111) plane of the Fd3m spinel phase. A careful examination of the HR-TEM of Zn-rich ZnAl_0.8_Fe_1.2_O_4_ in Figure 2e exposes the presence of a matrix corresponding to the Fe_2_O_3_ and Al_2_O_3_ phases. The SAED patterns in Figure 2A–E provide important insights into the crystal structures; where *x* = 0.0 wt.%, the diffraction spots are indicative of a well-defined crystalline structure. The rings in the pattern correspond to specific planes of the spinel structure. In Figure 2B, for the ZnAl_0.2_Fe_1.8_O_4_/polypyrrole composite, the diffraction spots are slightly less sharp than in Figure 2A, indicating a minor structural disorder. For Figure 2C–E, the SAED pattern shows more pronounced rings, suggesting an increase in structural disorder. The presence of rings alongside spots indicates that the samples contain regions of crystallinity as well as areas with less order, possibly due to the presence of secondary phases.

### 3.3. Magnetic Properties

**Figure 3:**
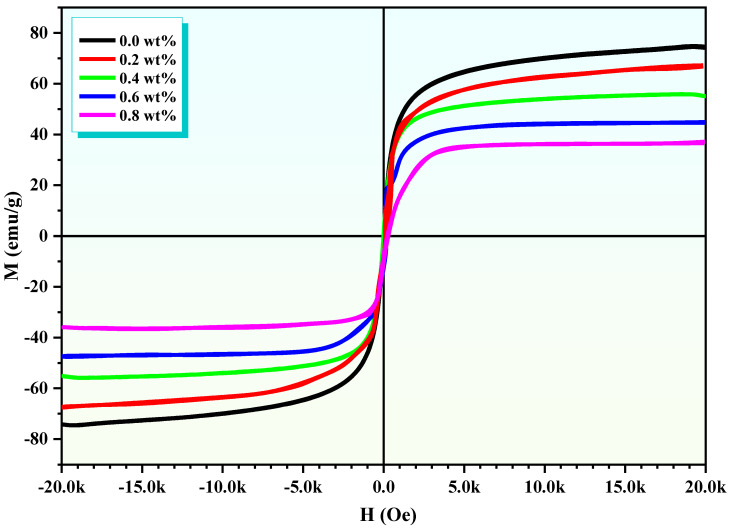
The hysteresis loops for ZnAl*_x_*Fe_2−*x*_O_4_/polypyrrole nanocomposite at *x* = 0.0, 0.2, 0.4, 0.6, and 0.8 wt.%.

Figure 3 shows the magnetic hysteresis loop for the ZnAl*_x_*Fe_2−*x*_O_4_/polypyrrole nanocomposite, with key magnetic properties at ambient temperature, such as loop area, saturation magnetization (Ms), remanence magnetization (Mr), the ratio of Mr to Ms (squareness), and coercivity (Hc), presented in Table 2. Given the polycrystalline nature of the structure with a size under 10 nm, it is likely to possess a mono-domain configuration, facilitating alignment with the external magnetic field with relative ease [17]. A notably low coercivity of 124.53 KOe suggests minimal resistance to domain alignment, with the unsaturated magnetization further implying a singular domain structure. The minimum value of Mr/Ms composites superparamagnetic-like properties. Polypyrrole, within this context, acts as a barrier to domain alignment under an external magnetic field but facilitates domain rotation when the field’s strength increases. The composite film’s squareness value, standing at a mere 0.106, reflects its soft magnetic ferrite nature [18]. The incorporation of polypyrrole, particularly at an *x* value of 0.6, degrades the magnetic attributes of the composites, leading to decreased saturation and remanence magnetizations. Specifically, the Ms and Mr readings for *x* = 0.6 are markedly reduced, registering at 0.4575 emu/g and 48.75 × 10^−3^ emu/g, respectively, an effect attributed to the diminished A-B exchange interaction caused by polypyrrole.

## 4. Dielectric and Microwave Properties 

**Figure 4:**
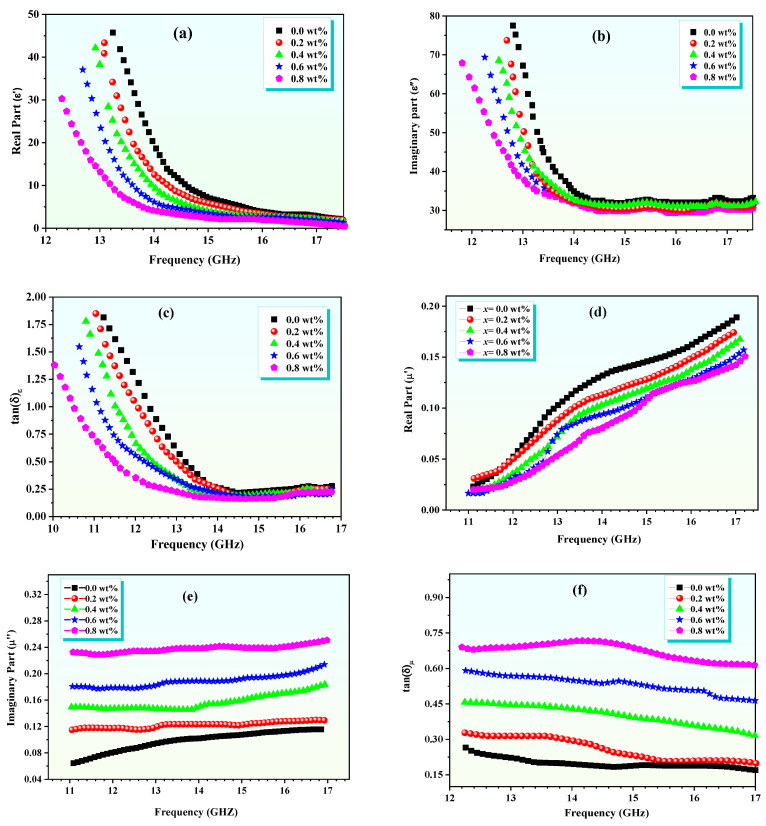
Frequency dependence of (**a**) real part of complex permittivity; (**b**) imaginary part of complex permittivity; (**c**) tan (δ)_ε_ for ZnAl*_x_*Fe_2−*x*_O_4_/polypyrrole nanocomposite; (**d**) real part of complex permeability; (**e**) imaginary part of complex permeability; (**f**) tan (δ)_μ_ for ZnAl*_x_*Fe_2−*x*_O_4_/polypyrrole nanocomposite at *x* = (0.0, 0.2, 0.4, 0.6, 0.8) wt.%.

The dielectric properties of the composite depend on the frequency subjected, which is closely linked to the polarizability and orientation of the present dipoles. As the frequency of the applied field increases, the dipoles of the system struggle to match the changing field faster, giving decline to the real and imaginary parts of the dielectric constant (ε′) and (ε″), respectively. The value of ε′ represents the strength of the dielectric polarization, which indicates the ability of the material to store electricity, while ε″ indicates the energy transfer to the dielectric material [19]. These values vary with the dielectric loss tangent (tan δ = ε″/ε′) at different frequencies. The frequency-dependent dielectric response of polypyrrole/Zn-Al ferrite nanocomposites with different Al content, as shown in Figure 4, was measured at ambient temperature in the frequency range of 10–17 GHz. The observation of a dielectric diffusion pattern shows a significant decrease in the values of ε′ and ε″, especially in the range of 10–15 GHz [20]. From this point on, ε′ decreases linearly up to 17 GHz, while ε″ and tan δ remain constant, unaffected by frequency changes. The initial phase decreases ε′ and ε″ are mainly due to the frequency elevation affecting the interface polarization, which is caused by charge accumulation at the interfaces between the conducting and dielectric blocks under an alternating electric field. Moreover, charge hopping plays an important role in Zn-Al ferrite. Nanostructures, with a large surface area compared to their size, exhibit numerous defects, dipoles, and unpaired bonds, enhancing the dipolar electron polarization effect and thus enhancing interface polarization. Generally, Zn-Al ferrite nanoparticles exhibit higher ε′, ε″, and tan δ values compared to polypyrrole/Zn-Al ferrite nanocomposites. ZnAl*_x_*Fe_2−*x*_O_4_/polypyrrole composites, characterized by a dense granular structure coated with polypyrrole chains on ferrite particles, exhibit variable electrical properties due to interactions between ferrite and polypyrrole to initiate polymerization, which can be degraded polymer chains and block electrical conductivity [21]. The encapsulation of ferrite nanoparticles in polypyrrole lowers the electron density, resulting in decreased conductivity. The addition of ZnAl ferrite to the polypyrrole matrix creates an additional space charge at the interface. Increasing the amount of Al increases the number of free valence electrons, which affects the conductivity properties of the composite. Figure 4c,d presents the frequency-dependent behavior of the electric modulus components, μ′ and μ″, along with their representation on a Cole–Cole plot (M′ versus M″). The M′ component showcases a progressive increase up to a frequency of *f* = 17 GHz, with enhanced values for the sample enriched with a higher Al concentration. Notably, at lower frequencies, the M′ values are non-zero, suggesting that the diminished ε′ values in these samples are not influenced by electrode polarization (EP) effects. There’s a clear correlation observed between the rising M′ values and the decreasing ε′ values. Space charge accumulation within electrical insulation emerges as a significant issue, as it amplifies the internal electric fields, potentially leading to partial electric discharges or dielectric breakdown, a process intricately linked to charge migration and storage [22]. The extraction of permittivity (ε) and permeability (μ) from S-parameters is indeed complex, as it depends heavily on the extraction model used. We have now explicitly described the NRW (Nicolson–Ross–Weir) method utilized for the extraction of ε and μ values from the measured S-parameters. Additionally, we have provided a brief comparison with other methods, such as the NRW (Nicolson–Ross–Weir) method [23] and the Boughriet method [24,25], to highlight the robustness and limitations of our chosen model. The sensitivity of the extracted parameters to the model selection has been discussed, acknowledging that different models could yield varying results. However, the NRW method is widely recognized for its reliability in the microwave frequency range, which justifies its use in this study.

The M″ component delineates two distinct relaxation peaks across different frequency bands. The initial peak, found in the lower frequency domain, underscores the dominant role of interfacial polarization (IP) relaxation phenomena attributed to charge build-up at the interfaces between the polypyrrole framework and the Zn-Al ferrite particles within the core–shell configurations. The subsequent peak, observed at higher frequencies, is attributed to α-relaxation, indicative of the local dynamics of polymer chain segments in polypyrrole, particularly dipolar polarization. The intensity of IP peaks surpasses that of the α-relaxation peaks significantly.

In addition to the NRW method used in this study, other methods, such as the Boughriet method, are also commonly employed for extracting permittivity and permeability from S-parameters. The Boughriet method, while effective in certain contexts, has limitations in terms of its accuracy at higher frequencies and for materials with complex magnetic properties. In contrast, the NRW method is widely recognized for its robustness and reliability, particularly in the microwave frequency range, making it a suitable choice for our study. However, it is important to note that the choice of extraction method can significantly impact the results, and care must be taken to select a method that aligns with the specific characteristics of the material under investigation.

The choice between these methods can significantly influence the results obtained in material characterization. The NRW method’s robustness makes it a preferred option for studies involving materials in the microwave frequency range, while the Boughriet method may be more applicable in situations where its specific advantages can be leveraged.

The Cole–Cole plot exhibits a semi-circular trajectory, deviating from the Debye model, followed by a linear increase. This semicircle’s radius inversely relates to the composite’s electrical conductivity; a larger radius suggests reduced conductivity [26]. The relaxation times (τIP) for samples with *x* = 0.0 wt.% and 0.2 wt.% are measured at 12,160.33 ms and 17,054.75 ms, respectively, illustrating the significant influence of Al^3+^ content on τIP values. The τIP values for Zn-Al ferrite samples are 816 ms and 2289 ms for grain boundary relaxation and 6.19 ms and 41 ms for grain relaxation, respectively. However, the τIP values for *x* = 0.4, 0.6, and 0.8 wt.% polypyrrole/Zn-Al ferrite nanocomposites are substantially higher, showing an increase of 7 to 15 times that of the grain boundary relaxation time, highlighting the profound effect of incorporating Al^3+^ into the matrix.

**Figure 5:**
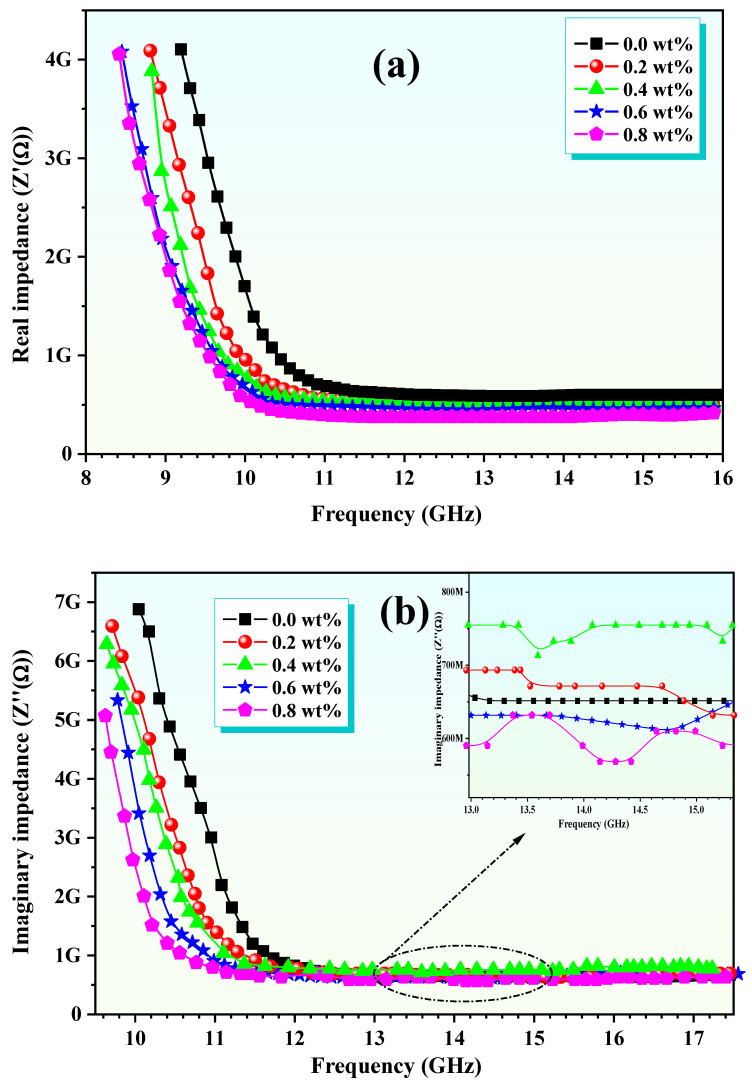
Frequency dependence of (**a**) the real impedance spectra and (**b**) the imaginary impedance spectra for ZnAl*_x_*Fe_2−*x*_O_4_/polypyrrole nanocomposite at *x* = 0.0, 0.2, 0.4, 0.6, and 0.8 wt.%.

The impedance spectroscopy has been utilized to explore the electrical characteristics of Zn-Al ferrite/polypyrrole nanocomposites, focusing on the analysis of changes in both the real (Z′) and imaginary (Z″) components of the impedance across different frequencies. These variations are graphically represented in a Cole–Cole plot, as illustrated in Figure 5, with measurements conducted at room temperature. To comprehend the dynamics between Z′ and Z″ over the entire frequency spectrum, it is essential to consider the behaviors of ε′ and ε″. Integrating ferrite nanoparticles within the polypyrrole matrix markedly reduces both Z′ and Z″ in the lower frequency domain (11–13 GHz), affecting the polymer matrix’s polymerization state and thus disrupting the continuity of its chains. This disruption leads to the emergence of interfacial polarization [24]. Nevertheless, as frequency increases, the significance of interfacial polarization wanes, giving way to the prominence of the charge-hopping mechanism within the Zn-Al ferrite. Between frequencies of 14 and 17 GHz, both Z′ and Z″ demonstrate stability, suggesting that the conductivity of polypyrrole predominates without interference from Zn ferrite, thus establishing a stable conduction pathway. The Cole–Cole plot reveals a semicircle that characterizes the Debye relaxation phenomena, essential for understanding the attenuation of electromagnetic waves. Z′ is indicative of the dissipation component, which accounts for the energy conversion from the input field to thermal energy, subsequently resulting in loss. On the other hand, Z″ represents the impedance’s imaginary component, reflecting the non-dissipative segment where the system temporarily stores the energy derived from the field, mimicking an ideal capacitor scenario. The semicircle displayed on the Cole–Cole plot is indicative of various Debye relaxation processes arising due to the interfacial polarization between the Zn-Al ferrite nanoparticles and the conductive polypyrrole matrix, leading to a textbook example of Debye relaxation. The impedance, or Nyquist diagram, delineates the relationship between Z′ and Z″ across varying frequencies (Figure 5), revealing a steady linear escalation across the full frequency range. This trend suggests a direct relationship between the increase in the dissipative component’s ratio and the impedance, which denotes the non-dissipative component. With an increase in frequency, there is a reduction in the energy loss, implying a decrease in the energy transition from the input field to the thermal reservoir, thereby allowing more energy to be conserved within the system.

The potential applications of Zn-Al/polypyrrole nanocomposites in advancing microwave application efficiency are promising due to their excellent microwave absorption performance. The synergistic effects of magnetic losses and dielectric losses contribute to their efficient electromagnetic wave absorption properties [27]. For instance, ZnFe_2_O_4_/polypyrrole nanocomposites exhibit broadband electromagnetic wave absorption at 8–18 GHz, with magnetic losses being the main microwave absorption mechanism [28]. Similarly, ZnFe_2_O_4_/SiO_2_/polypyrrole nanocomposites show efficient electromagnetic wave absorption properties in the K and Ka band regions, with magnetic losses and dielectric losses being the primary microwave absorption mechanisms [29]. In addition, the nanocomposites of polypyrrole with Zn-Al-ferrite exhibit a core–shell structure, and the possible bonding effect between metal cations and PPy results in a decrease in conductivity [30]. Microwave measurements are essential for evaluating the electromagnetic interference (EMI) shielding effectiveness (SE) of materials. The SE refers to the ability of a shielding material to attenuate or reduce the propagation of electromagnetic waves. It quantifies the level of attenuation provided by the shielding material and is expressed using Equations (1) and (2):

(4) $$SE(dB)=-10\log\left(\frac{P_{t}}{P_{i}}\right)$$

(5) $$SE_{T}=SE_{R}+SE_{A}+SE_{M}$$

where *P_i_* represents the power of the incident electromagnetic wave, and *P_t_* represents the power of the transmitted electromagnetic wave [31,32,33]. The total effectiveness of the electromagnetic shielding is being measured, by comparing the power of the electromagnetic waves before they encounter the shielding (the incident power, *P_i_*) versus the power of the waves that are able to transmit through the shielding (the transmitted power, *P_t_*). For a shielding material, the total shielding effectiveness is the sum of the contribution due to reflection (*SE_R_*), absorption (*SE_A_*), and multiple reflections (*SE_M_*). From the scattering parameters S_11_ and S_21_ of a vector network analyzer (measured by waveguide transmission line technique), the reflection coefficient (R) and transmission coefficient (T) can be obtained as $R={\left|S_{11}\right|}^{2}$ and $T={\left|S_{21}\right|}^{2}$. Using the parameters R and T, the absorption coefficient (A) can be evaluated as A + R + T = 1 [34,35]. With negligible multiple reflections between both interfaces of the material, the relative intensity of the effectively incident EM wave is based on the factor (1 − R), and the effective absorption can be expressed as A_eff_ = (1 − R − T)/(1 − R). The reflection and effective absorption can be expressed in decibels as *SE_R_* and *SE_A_* [36,37,38,39,40,41]:

(6) $$SE_{R}=10\log\left(1-R\right)$$

(7) $$SE_{A}=10\log\left(1-A_{eff}\right)=10\log\left(\frac{T}{1-R}\right)$$

**Figure 6:**
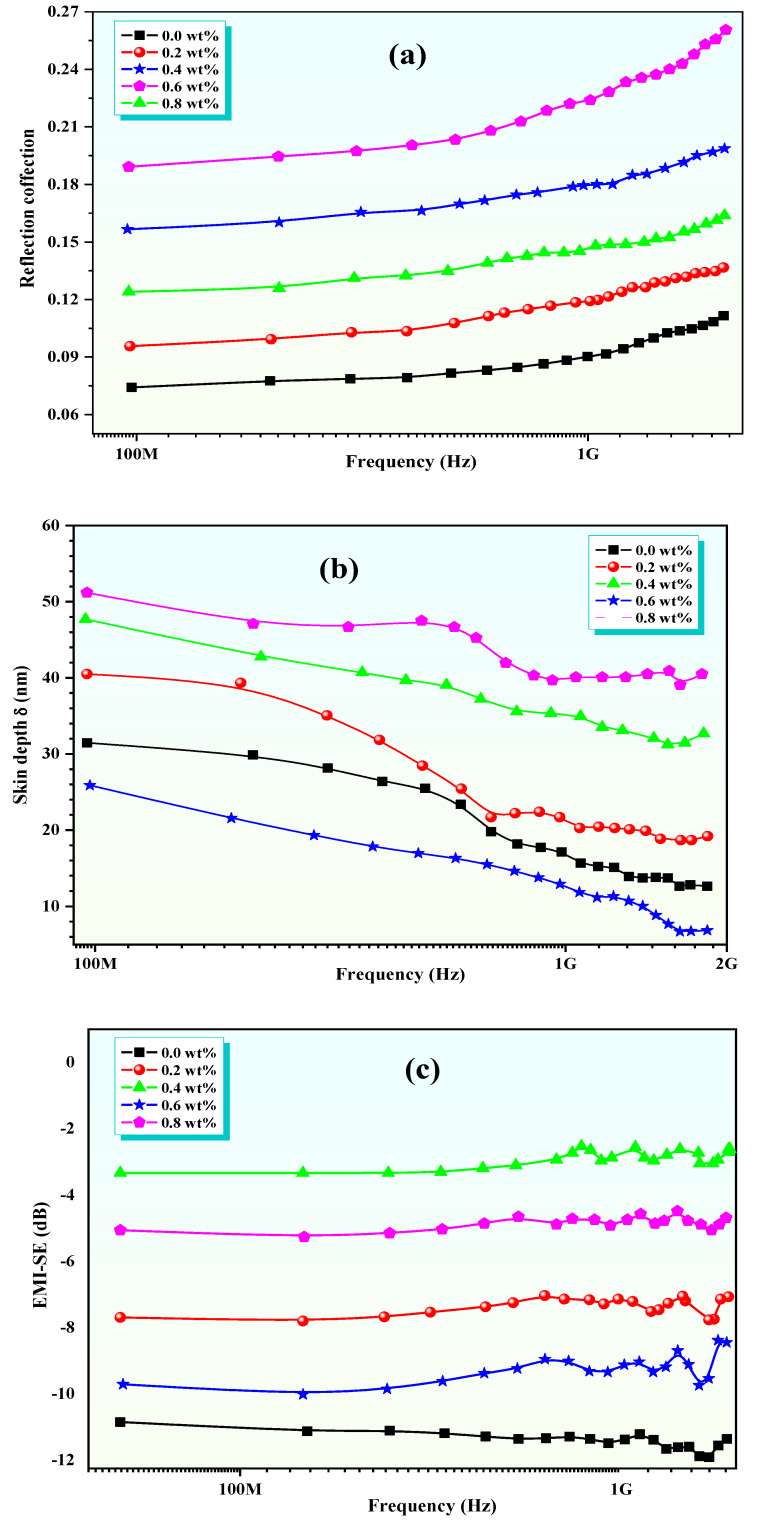
(**a**) Reflection coefficient, (**b**) skin depth, and (**c**) EMI-SE as a function of frequency for ZnAl*_x_*Fe_2−*x*_O_4_/polypyrrole nanocomposite at *x* = 0.0, 0.2, 0.4, 0.6, and 0.8 wt.%.

When negligible multiple reflections occur between the interfaces of the material, the relative intensity of the effectively incident electromagnetic wave can be estimated using the factor (1 − R). This factor determines the effective absorption of the material (Figure 6). 

## 5. Conclusions

This study presents the successful synthesis and comprehensive characterization of ZnAl*_x_*Fe_2−*x*_O_4_//polypyrrole nanocomposites with varying Al content (*x* = 0.0, 0.2, 0.4, 0.6, 0.8 wt.%). The addition of Al led to significant changes in both structural and magnetic properties, notably reducing the crystal size from 31 nm to 19.6 nm and decreasing the saturation magnetization (Ms) from 75 emu/g to 36 emu/g. The core–shell morphology observed in TEM analysis significantly influenced the dielectric behavior, with both real (ε′) and imaginary (ε″) dielectric constants decreasing as the frequency increased.

The dielectric properties, coupled with the results from impedance spectroscopy, underscore the role of interface polarization, with the relaxation time being notably affected by the Al content. The stability of both Z′ and Z″ within the 14–17 GHz frequency range suggests that the conductivity of polypyrrole predominates without interference from the Zn ferrite, making these nanocomposites particularly promising for microwave absorption applications. Furthermore, the electromagnetic parameters, ε and μ, extracted using the Nicolson–Ross–Weir method, confirmed the material’s potential for advanced microwave technologies. The combination of enhanced magnetic–dielectric characteristics points to Zn-Al ferrite/polypyrrole nanocomposites as viable candidates for applications in telecommunications, electromagnetic interference shielding, and other microwave-related technologies.

The Zn-Al ferrite/polypyrrole nanocomposite presents a promising material with enhanced magnetic and dielectric properties compared to previously studied materials. Its unique combination of properties makes it suitable for a wide range of applications, including magnetic data storage, microwave devices, and energy storage devices.

## Figures and Tables

**Table 1:** Crystalline size, D (nm); microstrain, ε; and dislocation, δ (nm^−3^).

*x* (wt.%)	D (nm)	ε	δ (nm^−3^)
0.0	30.01	0.00072	0.00111
0.2	27.13	0.00107	0.00136
0.4	23.90	0.00135	0.00175
0.6	21.02	0.00169	0.00226
0.8	19.89	0.00175	0.00253

**Table 2:** Saturation magnetization (Ms), remanent magnetization (Mr), coercivity (Hc), (Mr/Ms), and magnetic moment (μB) of ZnAl*_x_*Fe_2−*x*_O_4_/polypyrrole nanocomposite with concentrations of *x* (wt.%) = 0.0, 0.2, 0.4, 0.6, and 0.8.

*x* (wt.%)	Ms (emu/g)	Mr (emu/g)	Hc (Oe)	Mr/Ms	μB
0.0	75	2.26	38.91	0.030	10.79
0.2	67	3.87	38.52	0.057	9.17
0.4	55	2.19	38.86	0.039	7.15
0.6	45	1.48	39.17	0.032	5.54
0.8	36	2.00	39.64	0.055	4.18

## Data Availability

The authors confirm that the data supporting the findings of this study are available within the article.
